# Fine‐mapping and QTL tissue‐sharing information improves the reliability of causal gene identification

**DOI:** 10.1002/gepi.22346

**Published:** 2020-09-10

**Authors:** Alvaro N. Barbeira, Owen J. Melia, Yanyu Liang, Rodrigo Bonazzola, Gao Wang, Heather E. Wheeler, François Aguet, Kristin G. Ardlie, Xiaoquan Wen, Hae K. Im

**Affiliations:** ^1^ Section of Genetic Medicine, Department of Medicine The University of Chicago Chicago Illinois; ^2^ Department of Human Genetics The University of Chicago Chicago Illinois; ^3^ Department of Biology Loyola University Chicago Chicago Illinois; ^4^ Department of Computer Science Loyola University Chicago Chicago Illinois; ^5^ Department of Public Health Sciences, Stritch School of Medicine Loyola University Chicago Maywood Illinois; ^6^ The Broad Institute of MIT and Harvard Cambridge Massachusetts; ^7^ Department of Biostatistics University of Michigan Ann Arbor Michigan

**Keywords:** GWAS, PrediXcan, QTL integration

## Abstract

The integration of transcriptomic studies and genome‐wide association studies (GWAS) via imputed expression has seen extensive application in recent years, enabling the functional characterization and causal gene prioritization of GWAS loci. However, the techniques for imputing transcriptomic traits from DNA variation remain underdeveloped. Furthermore, associations found when linking eQTL studies to complex traits through methods like PrediXcan can lead to false positives due to linkage disequilibrium between distinct causal variants. Therefore, the best prediction performance models may not necessarily lead to more reliable causal gene discovery. With the goal of improving discoveries without increasing false positives, we develop and compare multiple transcriptomic imputation approaches using the most recent GTEx release of expression and splicing data on 17,382 RNA‐sequencing samples from 948 post‐mortem donors in 54 tissues. We find that informing prediction models with posterior causal probability from fine‐mapping (*dap‐g*) and borrowing information across tissues (*mashr*) can lead to better performance in terms of number and proportion of significant associations that are colocalized and the proportion of silver standard genes identified as indicated by precision‐recall and receiver operating characteristic curves. All prediction models are made publicly available at predictdb.org.

## 1 INTRODUCTION

Transcriptome studies with whole‐genome interrogation characterize genetic effects on gene expression traits. These mechanisms help elucidate the function of loci identified in genome‐wide association studies (GWAS) by identifying potential causal genes that link genetic variation with complex traits (Aguet et al., 2019; Albert & Kruglyak, 2015; Gusev et al., 2018; Huckins et al., 2019; Mancuso et al., 2018).

In particular, the Genotype‐Tissue Expression (GTEx) Project (Aguet et al., 2019) has sequenced whole genomes from 948 organ donors and generated RNA‐seq data across 52 tissues and 2 cell lines. Results and tools derived from this comprehensive catalog of transcriptome variation have enabled a myriad of applications such as drug repurposing (So et al., 2017) and clinical discoveries in cancer susceptibility genes (Wu et al., 2018), to name a few.

The general consensus that many noncoding variants associated with complex traits exercise their action via gene expression regulation has motivated the development of imputed transcriptome association approaches such as PrediXcan (Barbeira et al., 2018; Gamazon et al., 2015), TWAS/FUSION (Gusev et al., 2016), and UTMOST (Hu et al., 2019). In essence, these methods predict gene expression traits based on individuals' genotypes and test how these predictions correlate with complex traits.

Reliable prediction models for gene expression traits are key components of imputed transcriptome association studies. Given the predominantly sparse genetic architecture of gene expression traits (Wheeler et al., 2016) and overall robustness and performance (Fryett, Inshaw, Morris, & Cordell, 2018; Huckins et al., 2019), Elastic Net (Friedman, Hastie, & Tibshirani, 2010) has become the algorithm of choice for predicting transcriptome variation.

Despite Elastic Net's many advantages such as robustness and sparsity, we hypothesized that transcriptome imputation can be improved by leveraging biologically informed methods. Recent efforts (Hu et al., 2019) have exploited the high degree of eQTL sharing across tissues (Aguet et al., 2017) by leveraging cross‐tissue patterns in the broad GTEx panel to improve prediction performance, more notably in tissues with small sample sizes. Also, important methodological progress in fine‐mapping (Wang, Sarkar, Carbonetto, & Stephens, 2018; Wen, Pique‐Regi, & Luca, 2017) and an adaptive shrinkage method that improves effect size estimates across multiple experiments (Urbut, Wang, Carbonetto, & Stephens, 2019) provide opportunities to further improve quality of downstream associations.

In this article, we analyze different transcriptome prediction strategies and compare their strengths both in prediction performance and downstream phenotypic associations.

Proximity and linkage disequilibrium (LD) between distinct causal variants can lead to noncausal associations between predicted expression and complex traits (Barbeira et al., 2018; Wainberg et al., 2019). Since the ultimate goal of imputed transcriptome studies is to identify causal genes, our main focus here is to improve discoveries with less emphasis on expression prediction performance. We also applied the same model building techniques to alternative splicing traits quantified with LeafCutter (Y. I. Li et al., 2018). We make all results, prediction models and software available to the research community.

## 2 METHODS

We executed all methods using open source software running in a high‐performance cluster. We release all of our code and the data analyzed in this paper to ease reproducibility and accessibility.

### 2.1 GTEx data processing

We downloaded GTEx data for version 8 release from dbGAP (Accession Number phs000424.v8.p1). This data arises from 17,382 RNA‐seq samples from 54 tissues of 948 post‐mortem subjects, aligned to the GRCh38 assembly. Primary and extended results generated by consortium members are available on the Google Cloud Platform storage accessible via the GTEx Portal (see URLs).

Eight hundred and ninety‐nine whole‐genome sequencing samples were analyzed, 68 of them at an average coverage of 30x on HiSeq200, and the rest on HiSeqX. Eight hundred and sixty‐six GTEx donors' samples were included in the downstream variant call files, after excluding one each from 30 duplicate samples and 3 donors. Among these, 838 subjects with RNA‐seq data were included for QTL mapping and analysis.

Whole transcriptome RNA‐Seq data were aligned using STAR (v2.5.3.a; Dobin et al., 2013). For the STAR index, GENCODE v26 was used with the sjdbOverhang 75 for a 76‐bp paired‐end sequencing protocol. Default parameters were used for RNA‐Seq by expectation maximization (RSEM) (see URLs; B. Li & Dewey, 2011) index generation. GTExutilized Picard (see URLs) to mark and remove potential polymerase chain reaction (PCR) duplicates and RNA‐SeQC (DeLuca et al., 2012) to process postalignment quality control. RSEM was then used for per‐sample transcript quantification. Subsequently, read counts were normalized between samples using trimmed mean of M values (Robinson & Oshlack, 2010). For eQTL analyses, latent factor covariates were calculated using probabilistic estimation of expression residuals (PEER) (Stegle, Parts, Durbin, & Winn, 2010) as follows: 15 factors for *N* < 150 per tissue; 30 factors for 150 ≤ *N* < 250; 45 factors for 250 ≤* N* < 350; and 60 factors for *N* ≥ 350. Expression phenotypes were adjusted for unwanted variation using covariates such as gender, sequencing platform, and PCR protocol, the top five principal components from genotype data, and said PEER factors. Finally, fastQTL (Ongen, Buil, Brown, Dermitzakis, & Delaneau, 2016) was used for cis‐eQTL mapping in each tissue. Only protein‐coding, long intergenic noncoding RNA, and antisense biotypes as defined by Gencode v26 were considered for further analyses. To study alternative splicing, GTEx applied LeafCutter (version 0.2.8; Y. I. Li et al., 2018) using default parameters to quantify splicing QTLs in cis with intron excision ratios (Aguet et al., 2019).

We used the deterministic approximation of posteriors (*dap‐g*; Wen, Lee, Luca, & Pique‐Regi, 2016), *enloc* (Wen et al., 2017), and *coloc* (Giambartolomei et al., 2014) results published in Aguet et al. (2019).

### 2.2 GTEx expression and splicing modeling

We used the same genotypes, phenotypes, covariates, gene annotations, and variant annotations from the main GTEx analysis.

When building prediction models, we imposed an additional restriction: we used only samples of European ancestry for the sake of leveraging a well‐defined population LD structure. Only variants with minor allele frequency (MAF) < 0.05 in these samples were included. We used 49 tissues with sample sizes ranging from 65 (Kidney Cortex) to 602 (Muscle Skeletal).

This ancestry restriction mitigated problems due to LD mismatch when integrating with most publicly available GWAS summary statistics, which are conducted on predominantly European populations. Prediction models in other ancestries are important, and we are currently dedicating substantial effort to creating and analyzing such models. However, non‐European models are beyond the scope of this paper.

We only generated models for genes annotated in GENCODE v26 as protein‐coding, long noncoding RNA (lncRNA), or pseudogenes.

### 2.3 Elastic net models

We fitted an Elastic Net model for each gene–tissue pair with available adjusted expression data. We restricted the set of variants to those present in the HapMap 3 CEU track (Altshuler, Gibbs, Peltonen, & The International HapMap 3 Consortium, 2010) with MAF > 0.01. The motivation behind this choice was to restrict the analysis to a robust set of single nucleotide polymorphisms (SNPs) that has a significant intersection with the most publicly available GWAS summary statistics. For every gene, variants within 1 MB upstream of the gene's transcription start site and 1 MB downstream of the transcription end site where used as explanatory variables for gene expression.

We used the R package *glmnet* (Friedman et al., 2010), with mixing parameter *α* = .5 and penalty parameter chosen through 10‐fold cross‐validation.

Prediction performance was estimated using a nested cross‐validation approach. Expression was predicted out‐of‐sample for each fold, with Elastic Net parameters estimated only within training data, and the correlations to observed values at each fold were combined via Fisher's transformation and Stouffer's method. Only those models with mean Pearson's correlation across 10 folds *ρ* > .1 and nested cross‐validated correlation test *p* < .05 were kept.

We refer to these models as EN‐M.

### 2.4 Cross‐tissue gene expression imputation models

We employed the cross‐tissue gene expression imputation (CTIMP; Hu et al., 2019) framework on the same data from EN‐M models in the previous section. This method fits expression for a gene in multiple tissues simultaneously through a regularized linear model, using a Lasso penalty within each tissue and a group Lasso penalty for cross‐tissue patterns. As it internally uses genotypes from all samples available across all tissues, we expect improvements over EN‐M to be larger for tissues of smaller sample sizes where EN‐M deals with a less informative LD structure among variants.

We performed fivefold cross‐validation for model tuning and evaluation following the authors' description. We computed cross‐validated correlation measures across folds as in the previous method, and kept those models achieving the thresholds of cross‐validated correlation *ρ* > .1 and *p* < .05. As in EN‐M, we restricted the model training to variants in the HapMap 3 CEU track with MAF > 0.01; this became necessary because using all variants proved too computationally expensive since CTIMP consumes large amounts of memory and processing time. We briefly show in Figures S4–S6 that this additional restriction brings negligible effects in model training performance and prediction.

We refer to these models as CTIMP‐M.

### 2.5 Elastic net informed by *dap‐g* results

We also trained models via the Elastic Net algorithm using fine‐mapping information to refine the list of variants to be used as explanatory variables and lent more weight to variants with higher chances of affecting expression phenotypes. To this aim, we used *dap‐g*'s posterior inclusion probability (PIP) of a variant affecting gene expression to select explanatory variables, without restricting to variants in the HapMap CEU track. For every gene, we used all variants in the gene's cis‐window with MAF > 0.01 and PIP > 0.01. Since *dap‐g* groups variants in clusters according to LD, we kept the top variant (by PIP) per cluster to avert variable redundancy. Since we reasoned that more probable variants should bear more impact in the model's outcome, we multiplied each variant's penalty term in the Elastic Net regularization by a factor of 1 − PIP. We used the same thresholds from the previous subsections (*ρ *> .1 and *p* < .05) to select models with acceptable prediction performance.

We refer to these models as DAPGW‐M.

### 2.6 Multivariate adaptive shrinkage in R‐based models

Finally, we explored an entirely different algorithm to determine the prediction models. We executed multivariate adaptive shrinkage in R (*mashr*; Urbut et al., 2019) to estimate the models' effect sizes by leveraging cross‐tissue variations while allowing for sparse and possibly correlated effects in a Bayesian framework. We used *mashr* on the same set of variants from DAPGW‐M models. We kept models only for eGenes and effect sizes only for variants with PIP > 0.01 (from *dap‐g*) at each gene–tissue pair. Unfortunately, there is no natural prediction performance measure in this scenario as cross‐validation was not performed.

We refer to these models as MASHR‐M.

### 2.7 GEUVADIS data processing

We used GEUVADIS lymphoblastoid cell lines (LCL) expression study for independent validation of prediction performance. We obtained GEUVADIS expression data and sample information from the European Bioinformatics Institute web portal at https://www.ebi.ac.uk/. We obtained genotype data aligned to GRCh38 assembly from the International Genome Sample Resource web portal http://www.internationalgenome.org. We restricted data to individuals of European ancestry, yielding 341 samples.

For each one of the four previous model training schemes (EN‐M, CTIMP‐M, DAPGW‐M, and MASHR‐M) we predicted expression through PrediXcan (Gamazon et al., 2015) on GEUVADIS genotypes using GTEX LCL models and correlated predictions to observations.

### 2.8 GWAS processing and integration

We examined 87 GWAS from a heterogeneous set of traits first presented in the GTEx v8 study (Aguet et al., 2019; Barbeira, Bonazzola et al., 2019). These traits were selected to support a phenome‐wide study of the impact of gene regulation. Given the heterogeneous landscape of the GWAS, with intricate differences in data processing protocols and underlying human genome reference versions, it was necessary to make the GWAS variants homogeneous and compatible with those from the GTEx study.

First, the GWAS' variants were harmonized to the GTEx study's variants by mapping genomic coordinates via liftover (Haeussler et al., 2018; https://pypi.org/project/pyliftover) and keeping only variants with matching alleles. Then, GTEx variants with missing summary statistics for any GWAS were imputed with the best linear unbiased prediction method, a standard in the field (Lee, Bigdeli, Riley, Fanous, & Bacanu, 2013).

We executed S‐PrediXcan for each of four families of models (EN‐M, CTIMP‐M, DAPGW‐M, and MASHR‐M) using 49 tissues, for a total of 17,052 (trait, model family, and tissue) tuples. We integrated with *enloc* and *coloc* results published in Aguet et al. (2019).

When analyzing the versatility of the models and GWAS preprocessing schemes, we used GWAS studies not belonging to the rapid GWAS study. This was decided because the rapid GWAS project has a common, homogeneous variant set that could dominate comparisons.

### 2.9 AUC estimation for silver standard gene identification

**Figure 1:**
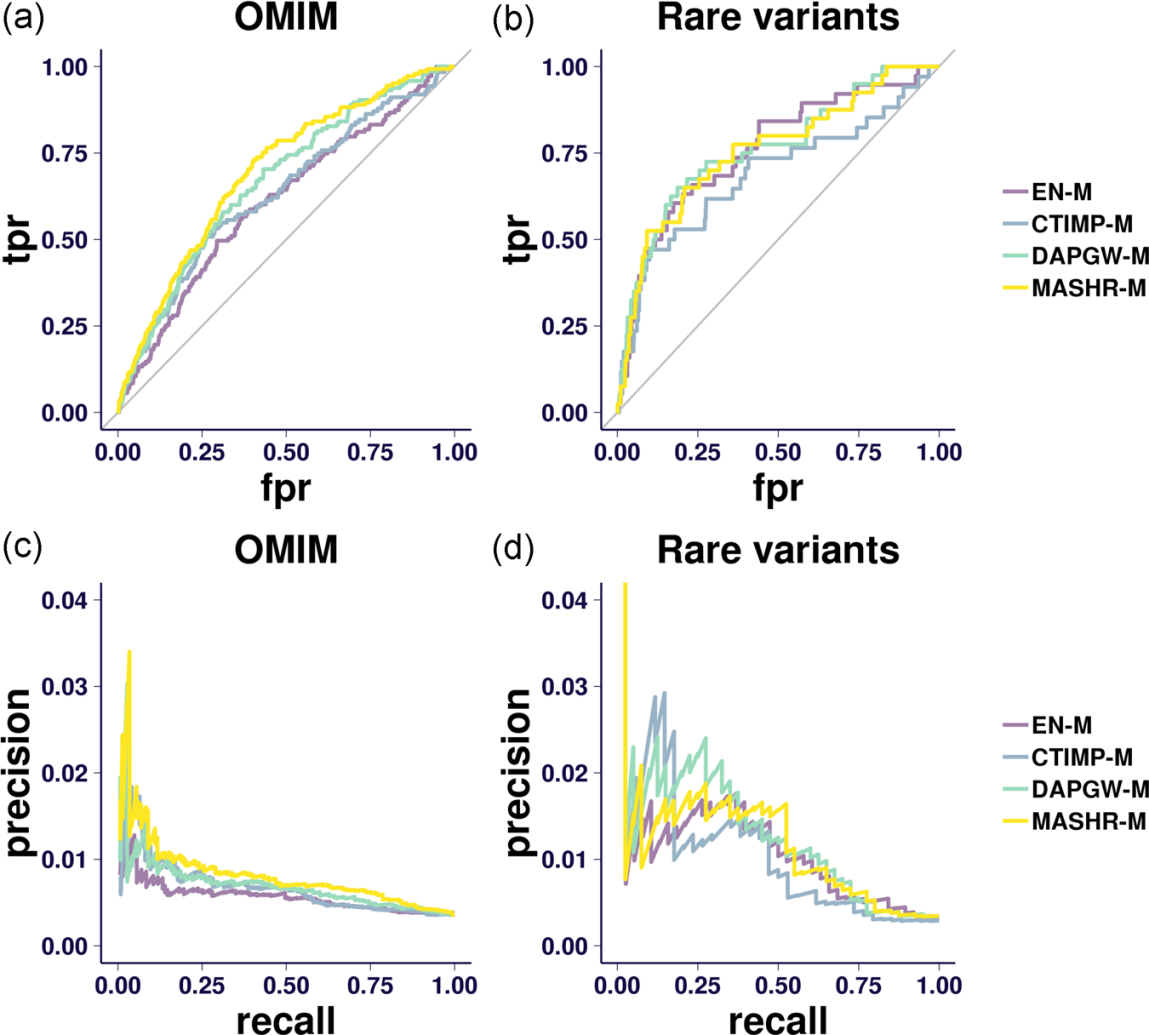
Receiver operating characteristic (ROC) and precision‐recall curve (PR) curves. (a) The ROC (plotting true‐positive ratio [TPR] to false‐positive ratio [FPR]) curve for the Online Mendelian Inheritance in Man (OMIM) silver standard. The area under the ROC curve (AUC) is greatest for the multivariate adaptive shrinkage in R (MASHR‐M) models, followed closely by the DAPGW‐M models. After using a bootstrap method to estimate a 95% confidence interval around the AUC estimates, we observe that the confidence intervals of all four model families overlap. (b) The ROC curve for the rare‐variant‐based silver standard. We observe that all strategies perform better than taking a random choice. However, this silver standard is too limited to properly distinguish between strategies. (c) The PR curve for the OMIM silver standard. MASHR‐M performs better than the other strategies in general but precision becomes a noisy measure towards lower recall ranges. (d) The PR curve for the rare‐variant‐based silver standard. The precision measure is too unstable to draw any conclusions. The OMIM silver standard not only validates the four proposed model strategies as a consistent approach to detect causal genes but provides additional evidence of MASHR‐M's superiority. The second silver standard, based on rare variants, is too limited to conclude anything beyond a high‐level validation of all four families

Figure 1a,b show the receiver operating characteristic (ROC) curve for silver standard gene identification in Online Mendelian Inheritance in Man (OMIM) and the rare‐variant‐based silver standard, respectively. To quantify the difference in performance among the different model families, we first computed the ROC of the two standards combined for each family. We then computed the area under the ROC curve (AUC) using the standard trapezoidal approach. The standard errors (*SE*s) of the estimated AUC were estimated by a bootstrap approach using 2,000 replicates, as implemented by Robin et al. (2011).

## 3 RESULTS

To identify optimal techniques for transcriptomic imputation, we have built models to predict genetically regulated expression using four different approaches on GTEx expression and splicing data (release version 8). To reduce LD misspecification problems, most apparent when applying summary statistics‐based versions of PrediXcan on GWAS of European populations, we used only European samples.

We restricted the analysis to genes that are annotated as protein‐coding, lncRNA, and pseudogenes in GENCODE version 26 (Frankish et al., 2019). We included 49 different tissues with sample sizes ranging from 65 (Kidney Cortex) to 602 (Muscle Skeletal).

The first strategy used the Elastic Net (Friedman et al., 2010) algorithm to compute predictions as described previously by Gamazon et al. (2015) and Wheeler et al. (2016). For every gene available in each tissue, this strategy used variants from the HapMap CEU track in a window ranging from 1 MB upstream of the transcription start site to 1 MB downstream of the transcription end site as explanatory variables. Only those models achieving thresholds of cross‐validated correlation (*ρ* > .1) and prediction performance *p* < .05 were kept. We will refer to this family as the **EN‐M** models.

The second strategy used CTIMP (Hu et al., 2019). CTIMP uses a regularized, generalized linear regression algorithm to fit expression from different tissues simultaneously. CTIMP optimizes a cost function including a within‐tissue Lasso penalty and a cross‐tissue group Lasso penalty, thus inheriting Lasso‐like behavior that is less sparse than Elastic Net. We used the same variants from the EN‐M strategy (HapMap CEU track, same windows around each gene), and identical correlation threshold (*ρ*  > .1) and cross‐validated prediction performance threshold (*p* < .05) to accept models. We will refer to this family as the **CTIMP‐M** family. We verified that this method's performance is not significantly improved by using all available GTEx variants, as explained in the Supporting Information Materials.

The third strategy used the PIP of a variant being causal for gene expression as estimated by the Bayesian fine‐mapping method *dap‐g* (Wen et al., 2016). First, for every gene, we restricted to variants with posterior inclusion probabilities PIP > 0.01. Since *dap‐g* clusters variants by their LD, we kept the variant with the highest PIP from each cluster to avoid redundant explanatory variables. Then, the selected variants were fed into the Elastic Net algorithm, scaling each variant's effect size penalty by a factor of 1 − PIP (i.e., more likely variants are less penalized). Only those models achieving good enough cross‐validated prediction performance (*p *< .05) and correlation (*ρ* > .1) were kept. We will refer to this family as **DAPGW‐M** (*dap‐g* weighted). As discussed later, the cross‐validated prediction performance of this approach cannot be fairly compared to EN‐M and CTIMP‐M because the preselection of fine‐mapped variants is based on the same underlying data.

The fourth strategy used *mashr* (Urbut et al., 2019) effect sizes from variants selected by *dap‐g* as in the **DAPGW‐M** approach. More specifically, fine‐mapped variants were selected as in the **DAPGW‐M** approach but the weights were obtained by applying *mashr* to the marginal effect sizes and *SE*s from the GTEx eQTL analysis (Aguet et al., 2019). Unlike the previous methods, this approach does not fit into a cross‐validation strategy and therefore lacks a natural prediction performance measure. Only eGenes with at least one cluster of variants achieving *dap‐g* PIP > 0.1 were kept. We will refer to this family as **MASHR‐M**.

We did not consider the BSLMM family of methods for transcriptome prediction. These models contain both a sparse and a polygenic component. The latter is likely to induce LD contamination (Barbeira et al., 2018) and does not reflect the sparse architecture of expression traits (Wheeler et al., 2016).

We also applied the EN‐M and MASHR‐M methods to alternative splicing quantification from LeafCutter (Y. I. Li et al., 2018) and made them readily available to the research community. These models were extensively used by Aguet et al. (2019) and Barbeira, Bonazzola et al. (2019).

### 3.1 Summary of models

**Figure 2:**
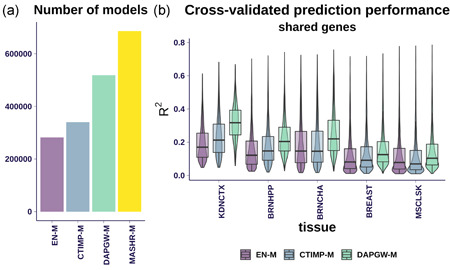
Models summary. (a) The number of models generated for protein‐coding genes, pseudogenes, and lncRNA across the four strategies. MASHR‐M displayed the largest number of generated models. (b) Compares prediction performances for gene–tissue pairs present in all four strategies, at five different tissues ordered by sample size. CTIMP‐M performed better than EN‐M in tissues with a smaller sample size. DAPGW‐M is presented for illustration purposes; since it included an additional variable selection step using the same underlying data, it cannot be fairly compared to EN‐M and CTIMP‐M. MASHR‐M does not have a prediction performance measure. The intersection of gene–tissue pairs across the four strategies is mostly defined by Elastic Net, the smallest set. 82% of Elastic Net models make up the intersection available to all strategies. Tissue abbreviations and sample size: KDNCTX: kidney—cortex, *n* = 65; BRNHPP: brain—hippocampus, *n* = 150; BRNCHA: brain—cerebellum, *n* = 188; BREAST: breast—mammary tissue, *n* = 337; MSCLSK: muscle—skeletal, *n* = 602. lncRNA, long noncoding RNA

Given the differences in the computational approach, not all prediction strategies generated models for every available gene–tissue pair. As can be seen in Figure 2a, EN‐M yielded the smallest number of valid models, for 281,848 gene–tissue pairs. CTIMP‐M produced 340,104 valid models, 21% more than EN‐M, as expected from its integration of multiple tissues' information.

Fine‐mapping‐based methods generated even more models: 518,537 from DAPGW‐M (84% more than EN‐M) and 686,241 from MASHR‐M (143% more than EN‐M). Please note that given the different criteria used to accept a model as valid, simple counts of available models should not be considered a measure of performance.

We show the distribution of cross‐validated prediction performances in Figure 2b We include five representative tissues ordered by increasing sample size (kidney, brain—hippocampus, brain—cerebellum, breast, and skeletal muscle). To perform a uniform comparison, we used only gene–tissue pairs available to all model families. CTIMP‐M showed better prediction performance than EN‐M on tissues with a smaller sample size but performed similarly on tissues with larger sample sizes. We attribute this to CTIMP's design, which leveraged all existing samples' genotypes in the tissues of smaller expression sample size. MASHR‐M models had no natural prediction performance measure and thus are excluded from these panels. DAPGW‐M is presented for completeness but its comparison to EN‐M and CTIMP‐M is unfair. We show in Figure S1 the cross‐validated prediction performances for all genes in each family.

### 3.2 Fine‐mapping‐based models perform well in the independent expression data set

**Figure 3:**
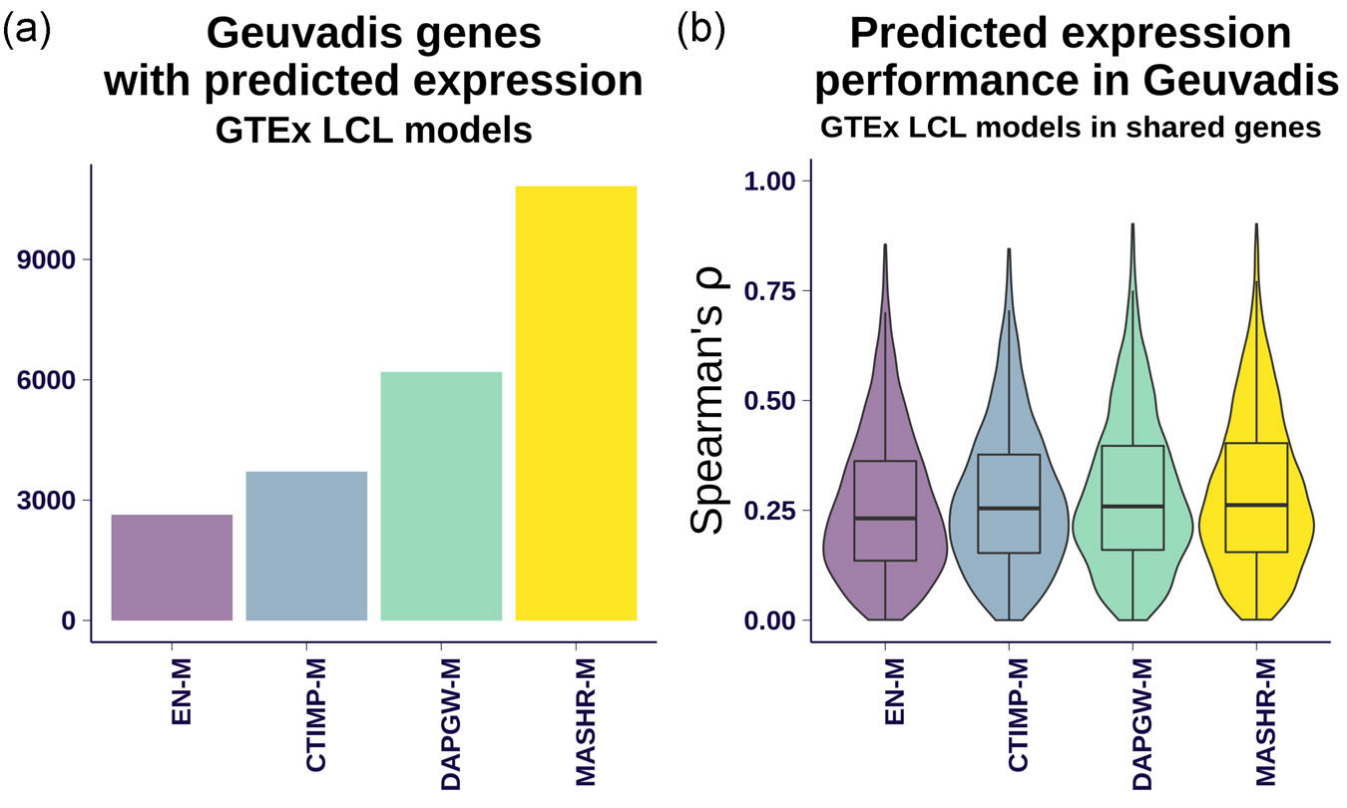
Validation in a separate expression cohort. (a) The number of genes predicted in the GEUVADIS cohort using the LCL models from each of the four strategies. MASHR‐M had the most models available, followed in decreasing order by DAPGW‐M, CTIMP‐M, and EN‐M. (b) The distribution of prediction performances (Spearman's ρ) for genes available to all four families. DAPGW‐M and MASHR‐M performed slightly but consistently better than EN‐M and CTIMP‐M. We attributed the small differences to the GTEx LCL tissue having a small sample size (*n* = 115 individuals), much lower than the 341 available in GEUVADIS. Also, the intersection of genes available to all four strategies is dominated by those present in Elastic Net, the smallest set; and genes that can be modeled with Elastic Net tend to be the ones with less complicated patterns of variation. GTEx, Genotype‐Tissue Expression Project; LCL, lymphoblastoid cell lines

Next, we sought to validate the models' predictions in an independent RNA‐seq data set. We analyzed data from the GEUVADIS project (Lappalainen et al., 2013), which includes 341 samples of European ancestry with genotype and LCL expression data. We predicted expression using GTEx LCL models from the four strategies and compared them with measured expression levels. Figure 3a shows the number of genes that each family was able to predict. DAPGW‐M and MASHR‐M had the largest number of predictable genes, followed by CTIMP‐M and EN‐M.

To compare prediction performances, we used Spearman's rank correlation coefficient ρ as a robust measure that handles the scale and complexity differences between real GEUVADIS expression data and predicted expression levels. Figure 3b shows the distribution of prediction performance (Spearman's ρ) for genes present in all four methods on the LCL tissue. We observed that all four families achieved similar levels of performance, with MASHR‐M, DAPGW‐M, and CTIMP‐M faring slightly but consistently better than EN‐M. Mean correlations were .028 (*SE* = 0.006), .027 (*SE* = 0.006), and .018 (*SE* = 0.006) points larger for MASHR‐M, DAPGW‐M, and CTIMP‐M, respectively compared to EN‐M.

We attribute the smaller performance differences to low power, since GTEx LCL tissue has a sample size of *n* = 115 individuals, much lower than the 341 available in GEUVADIS.

### 3.3 Fine‐mapping improves the number and colocalization of associations

Next, we assessed whether any of these models perform better at identifying causal genes. We considered the number and proportion of colocalized genes among the significant ones as measures of association quality.

We used the four families of models to correlate predicted expression with 87 phenotypes through 49 tissues using the summary version of PrediXcan. Results of applying the EN‐M models to GWAS summary statistics, harmonized and imputed to GRCh38 (Schneider et al., 2017), were presented by Aguet et al. (2019). In this section, we say that a gene–tissue pair is significant if it achieves a *p*‐value below the Bonferroni‐corrected threshold (0.05/number of gene–tissue pairs) within each trait.

We used *enloc* (Wen et al., 2017) results published in Aguet et al. (2019) to assess the colocalization status of GWAS and transcriptomic traits as evidence for a shared underlying mechanism. Briefly, *enloc* computes the “regional colocalization probability” (rcp) that a trait shares causal variants with a gene's expression (or an intron's splicing quantification), within a GWAS region and the overlapping gene's cis‐window. We say that a gene–tissue pair is “colocalized” with a trait if it achieves an *enloc* rcp > 0.5. Note that rcp ≤ 0.5 should not be interpreted as a false association; rather, it only means that there is not enough evidence of colocalization. See Section 4 on the conservative nature of colocalization approaches in Barbeira, Bonazzola et al. (2019).

We say that a gene–tissue pair that is both significant and colocalized is a “prioritized” detection or candidate. To simplify the interpretation of results across multiple tissues, we count the number of unique genes among the prioritized gene–tissue pairs for each trait.

**Figure 4:**
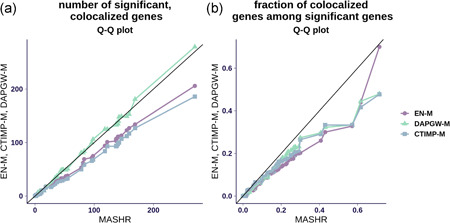
PrediXcan associations across 87 traits (a) A Q–Q plot for the number of colocalized, significant genes per trait. Fine‐mapping‐informed models (DAPGW‐M and MASHR‐M) achieved similar numbers of colocalized detections, both slightly higher than EN‐M and CTIMP‐M. (b) A Q–Q plot for the fraction of colocalized genes among significant genes per trait. MASHR‐M's distribution is shifted towards higher proportions than the other families. We say a gene is significant if it achieves a Bonferroni‐adjusted threshold of 0.05 per number of available gene–tissue pairs, in at least one tissue. Likewise, we say a gene is colocalized if it achieves *enloc* rcp > 0.5 in any tissue. We say a gene is a candidate or “prioritized” detection if it is both significant and colocalized in any tissue. rcp, regional colocalization probability

We found that MASHR‐M typically yields more candidate genes. On average 28.3 (standard deviation [*SD*] = 44.4), 25.5 (*SD* = 40.3), 36.7 (*SD *= 57.8), 36.6 (*SD* = 57.0) genes were identified with EN‐M, CTIMP‐M, DAPGW‐M, and MASHR‐M, respectively. We display the numbers of detections for each trait in Figure 4, through Q–Q plots comparing MASHR‐M to the other the model families. We observe in Figure 4a that the fine‐mapping informed families of models, DAPGW‐M and MASHR‐M, yielded a similar number of candidates per trait, consistently larger than EN‐M and CTIMP‐M. When comparing the fraction of colocalized genes among significant genes (Figure 4b), MASHR‐M yielded a larger proportion of colocalized genes compared to the other three families. On average 8.18% (*SE* = 0.013), 8.88% (*SE* = 0.013), 9.01% (*SE* = 0.013), 11.7% (*SE* = 0.013) of identified genes with EN‐M, CTIMP‐M, DAPGW‐M, and MASHR‐M, respectively, were colocalized. In general, we observed that associations obtained through both DAPGW‐M and MASHR‐M models tend to agree (see Figure S2 as an example).

We were thus led to favor MASHR‐M, which produced the largest number of models, with a larger number of colocalized, significant associations as well as higher proportions of colocalized associations among significant genes.

*Enloc* relies on the *dap‐g* algorithm itself as a component, so that the fraction of colocalized genes could have been biased towards *dap‐g* informed methods. To make sure that the use of *dap‐g* is not driving the improved colocalization rate of MASHR‐M over the other strategies, we verified the performance using another colocalization method, *coloc* (Giambartolomei et al., 2014).

We observed that MASHR‐M still had a better rate of colocalization among significant associations, although with smaller differences as can be seen in Figure S3. This is probably in part due to *coloc*'s reduced power and limiting assumption of a single causal variant (see Barbeira, Bonazzola et al., 2019 for details).

### 3.4 Fine‐mapping improves identification of silver standard genes

As an independent way to assess each prediction strategy's ability to identify causal genes, we framed the problem as one of causal gene prediction and use standard prediction performance measures such as ROC and precision‐recall. This avoids using an ad‐hoc significance or colocalization thresholds.

As proxies for causal genes, we leveraged two different “silver standards” as described by Barbeira, Bonazzola et al. (2019). The first one, based on the OMIM database (Amberger, Bocchini, Scott, & Hamosh, 2019), features 1,592 known gene–trait associations. The second one is based on rare‐variant association studies (Liu et al., 2017; Locke et al., 2019; Marouli et al., 2017) and contains 101 gene–trait associations.

We restricted our analysis to gene–trait pairs in the vicinity of the corresponding traits' GWAS loci since we did not expect any of the methods to detect reliable signals elsewhere. We used approximately independent LD regions (Berisa & Pickrell, 2016) to define the vicinity.

Using absolute values of *z* scores as an association scores for each strategy, we assessed their ability to “predict” the silver standard gene–trait associations. We show in Figure 1 the ROC and PR curves on OMIM and rare‐variant‐based silver standards.

Using the OMIM‐based silver standard (Figures 1a and 1c), we observed that the MASHR‐M strategy outperforms the other strategies, with DAPGW‐M a close second.

Using the rare‐variant‐based silver standard (Figures 1b and 1d), we observed that all four strategies are able to detect known causal genes. However, the limited size of this standard did not allow us to distinguish between the four families.

When considering the AUC for the combined OMIM and rare‐variant‐based silver standards, we computed the point estimate and estimated the *SE*s using a bootstrap approach (implemented in Robin et al., 2011). We observed the MASHR‐M models had the highest AUC of all of the model families. Differences in AUC between MASHR‐M and CTIMP‐M and EN‐M models were 0.0636 (*SE* = 0.0307) and 0.0682 (*SE* = 0.0287), respectively, providing evidence that MASHR‐M models are better equipped for detecting known genes and reinforcing our choice of MASHR‐M as the best option.

### 3.5 Importance of imputation of missing summary statistics in practice

The prediction models' usefulness depends on the availability of their variants in the GWAS of interest. Publicly available GWAS use different sequencing and genotyping techniques, based on different genotype imputation panels and human genome release versions so that the lists of available variants vary wildly across traits. Thus, a GWAS might lack particular variants from a prediction model, so that the model cannot properly infer variation patterns as shown in Barbeira, Pividori et al. (2019). Since many fine‐mapped variants in the GRCh38‐based GTEx study can be absent in a typical GWAS, we sought to assess the impact of variant compatibility in real applications.

We compared S‐PrediXcan results from MASHR‐M models on 69 publicly available GWAS with two preprocessing schemes:

1. Harmonization only (no imputation): Simple harmonization of variants by lifting over genomic coordinates from the GWAS to match the GRCh38‐based GTEx prediction models, and then filtering for matching alleles (“Harmonization” for short)
2. Harmonization and imputation of missing summary statistics (“Imputation” for short) on harmonized GWAS.

The 69 traits included in this analysis are those among the 87 traits not belonging to the Rapid GWAS project, to prevent the highly homogeneous Rapid GWAS datasets from dominating comparisons.

**Figure 5:**
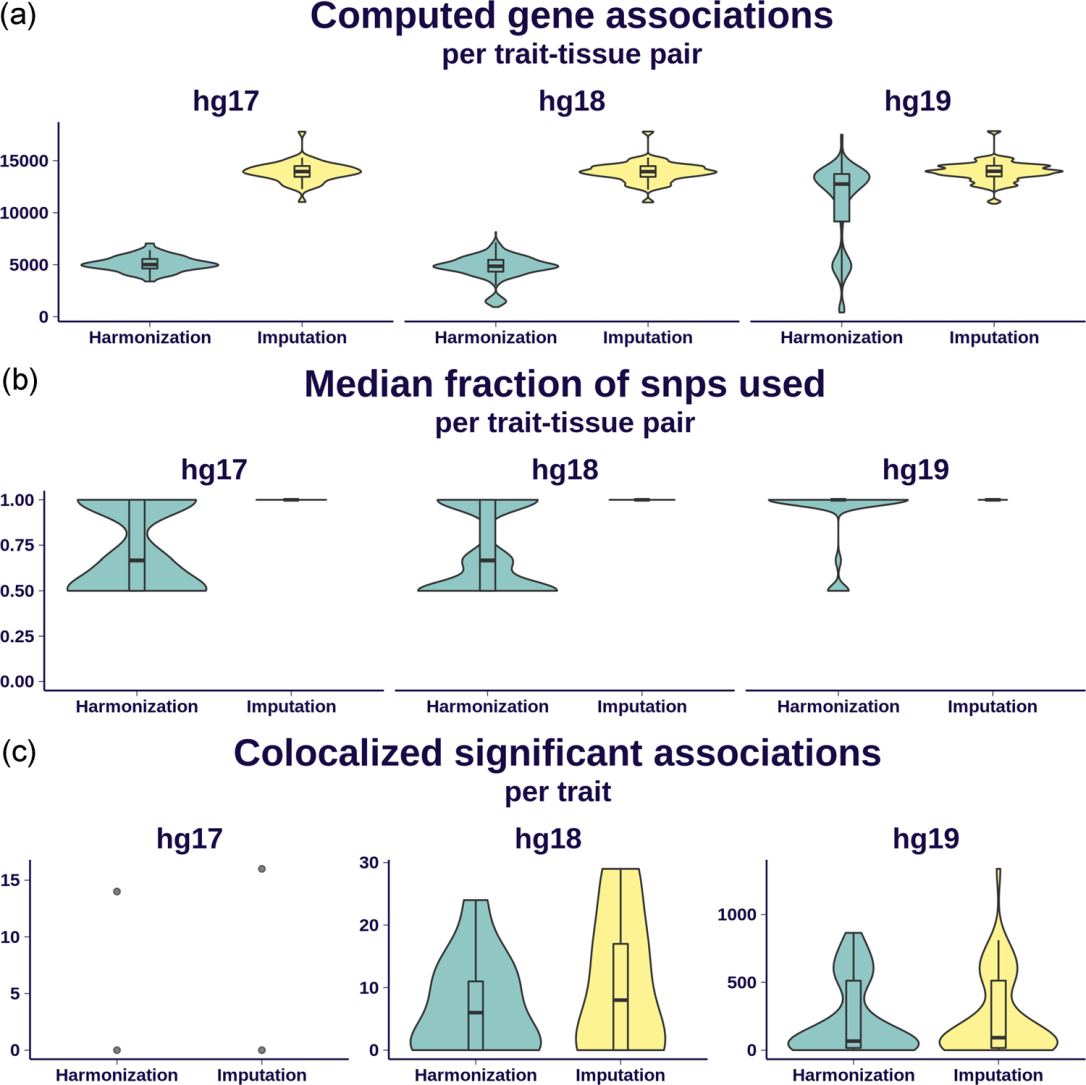
Effect of imputation on association quality. We display here a comparison of S‐PrediXcan results from MASHR‐M models on 69 GWAS traits using two different preprocessing schemes: simple harmonization of GWAS variants to GTEx's, and additional imputation of missing summary statistics. Results are grouped by the different human genome release versions underlying each GWAS: 2 traits were defined on hg17, 13 on hg18, and 54 on hg19. (a) The distribution of the number of associations per trait–tissue pair that can be computed; imputation dramatically increased the number of associations for hg17‐ and hg18‐based traits. Some hg19‐based traits exhibited a good number of computable associations after just a simple harmonization. Panel (b) shows, per trait–tissue pair, the distribution of the median fraction of model SNPs present in the GWAS. It is nearly one for the most trait–tissue pairs in the imputation scheme, ranging between 0.5 and 1 with the harmonization scheme. (c) The number of colocalized, significantly associated genes that can be found after applying imputation and harmonization schemes. The gain of imputation for hg19 is less dramatic than in the other comparisons in this figure, given the conservative nature of the colocalization filter. GTEx, genotype‐tissue expression; GWAS, genome‐wide association studies; SNP, single nucleotide polymorphism

We show in Figure 5 the effect of these preprocessing schemes on various performance metrics, segregated by the human genome release version (hg17, hg18, and hg19).

Figure 5a summarizes the increase in the number of gene associations computed for every trait–tissue pair. For hg17‐ and hg18‐based GWAS, the gain through summary‐statistics imputation is almost threefold. Some hg19‐based GWAS traits without imputation yield a good enough number of computable genes.

Figure 5b shows the distribution of the median fraction of model SNPs also present in the GWAS, within each tissue–trait combination. Roughly 60% of models' variants are present in hg17‐ and hg18‐based GWAS without imputation; this percentage is substantially higher for hg19‐based GWAS without imputation. Imputing summary statistics increase this median percentage to 100% on all tissue–trait combinations across the analyzed human genome release versions.

Figure 5c shows the increase in the number of genes detected per trait. As in the previous panels, the increase is more noticeable for hg17‐ and hg18‐based GWAS, while smaller for hg19‐based studies.

Therefore, we recommend to always perform variant harmonization due to its low complexity and time requirements, followed by summary‐statistics imputation if possible. For newer GWAS with modern sequencing and genotyping, summary‐statistics imputation may not be as critical depending on their intersection with model variants.

## 4 DISCUSSION

Through extensive analysis of different model training schemes, we conclude that using fine‐mapping information (from *dap‐g*) and cross‐tissue patterns (from *mashr*) improve the reliability of causal gene detection. These models (MASHR‐M) yield more detections when integrating GWAS and eQTL studies and show improved performance when validating results in a silver standard of known gene‐to‐trait associations (OMIM database). We make all prediction models and results publicly available.

Special consideration must be paid to how well each model's variants intersect GWAS' variants. Fine‐mapping‐informed models are sparse and parsimonious. This could be a hurdle when the fine‐mapped variants of import are missing or have low imputation quality in a GWAS, as is often the case with older studies. In this scenario, our recommendation is to impute any missing variants. If that is not possible, the association with the incomplete prediction may still detect the underlying association albeit with reduced power. The MASHR‐M and DAPGW‐M models have predictors that belong to different LD clusters and the effect sizes are based on marginal regression and smoothing across tissues such that missing one of the “causal clusters” is unlikely to add false positives. The alternative is falling back to models such as CTIMP‐M, defined on a robust set of variants available to most GWAS, at the cost of decreased performance (detection and prediction). EN‐M additionally features some “built‐in” redundancy: for a set of variants in LD among each other, they all tend to be included in a model with the effect spread between them.

While our recommended MASHR‐M method offers several benefits compared to existing approaches, there is still room for improvement. Potential developments could rely on fine‐mapping methods that jointly incorporate cross‐tissue patterns or consensus between different fine‐mapping approaches. Also, epigenetic information has been shown to improve transcriptome prediction (Zhang et al., 2019) as well. Future improvements should incorporate this epigenetic information and other biologically informed annotations jointly.

Our validation in silver standards, especially our difficulty interpreting the results from the rare‐variant‐based silver standard, also illustrates the need for well‐curated, large databases of known gene‐to‐phenotype associations to assess the performance of either new or improved methods.

In conclusion, we present here a method for predicting the genetically regulated component of transcriptomic traits with a superior performance both in terms of prediction performance and gene–trait association detection.

## CONFLICT OF INTERESTS

F. A. is an inventor on a patent application related to TensorQTL. S. E. C. is a cofounder, chief technology officer, and stock owner at Variant Bio. E. T. D. is chairman and member of the board of Hybridstat Ltd. B. E. E. is on the scientific advisory boards of Celsius Therapeutics and Freenome. G. G. receives research funds from IBM and Pharmacyclics, and is an inventor on patent applications related to MuTect, ABSOLUTE, MutSig, POLYSOLVER, and TensorQTL. S. B. M. is on the scientific advisory board of Prime Genomics Inc. D. G. M. is a cofounder with equity in Goldfinch Bio, and has received research support from AbbVie, Astellas, Biogen, BioMarin, Eisai, Merck, Pfizer, and Sanofi‐Genzyme. H. K. I. has received speaker honoraria from GSK and AbbVie. T. L. is a scientific advisory board member of Variant Bio with equity and Goldfinch Bio. P. F. is a member of the scientific advisory boards of Fabric Genomics Inc., and Eagle Genomes Ltd. P. G. F. is a partner of Bioinf2Bio. E. R. G. receives an honorarium from Circulation Research, the official journal of the American Heart Association, as a member of the Editorial Board, and has performed consulting for the City of Hope/Beckman Research Institute. R. D. has received research support from AstraZeneca and Goldfinch Bio, not related to this study.

## Supporting information

Supplementary Information

## Data Availability

Genotype‐Tissue Expression (GTEx) Project's raw whole transcriptome and genome sequencing data are available via dbGaP accession number phs000424.v8.p1. All processed GTEx data are available via the GTEx portal. Imputed summary results, *enloc, coloc*, PrediXcan, MultiXcan, *dap‐g*, prediction models, and reproducible analysis are available in https://github.com/hakyimlab/gtex-gwas-analysis and links therein. flashr, https://gaow.github.io/mnm-gtex-v8/analysis/mashr_flashr_workflow.html#flashr-prior-covariances; mashr, https://github.com/stephenslab/mashr; Gencode, https://www.gencodegenes.org/releases/26.html; GTEx GWAS subgroup repository, https://github.com/broadinstitute/gtex-v8; GTEx portal, http://gtexportal.org; Hail, https://github.com/hail-is/hail; MetaXcan, https://github.com/hakyimlab/MetaXcan; pyliftover, https://pypi.org/project/pyliftover/; Summary GWAS imputation, https://github.com/hakyimlab/summary-gwas-imputation; TORUS, https://github.com/xqwen/torus; DAP, https://github.com/xqwen/dap; and UK Biobank GWAS, http://www.nealelab.is/uk-biobank/;
