# Supplementary material for: Fine‐mapping and QTL tissue‐sharing information improves the reliability of causal gene identification
Source: Genet Epidemiol. 2020 Sep 10;44(8):854–67. Online ahead of print. doi: 10.1002/gepi.22346 (PMC7693040; doi:10.1002/gepi.22346)
Supplement: Supplementary file 1 — Supplementary Information [file GEPI-44-854-s001.pdf]

Supplementary Figures

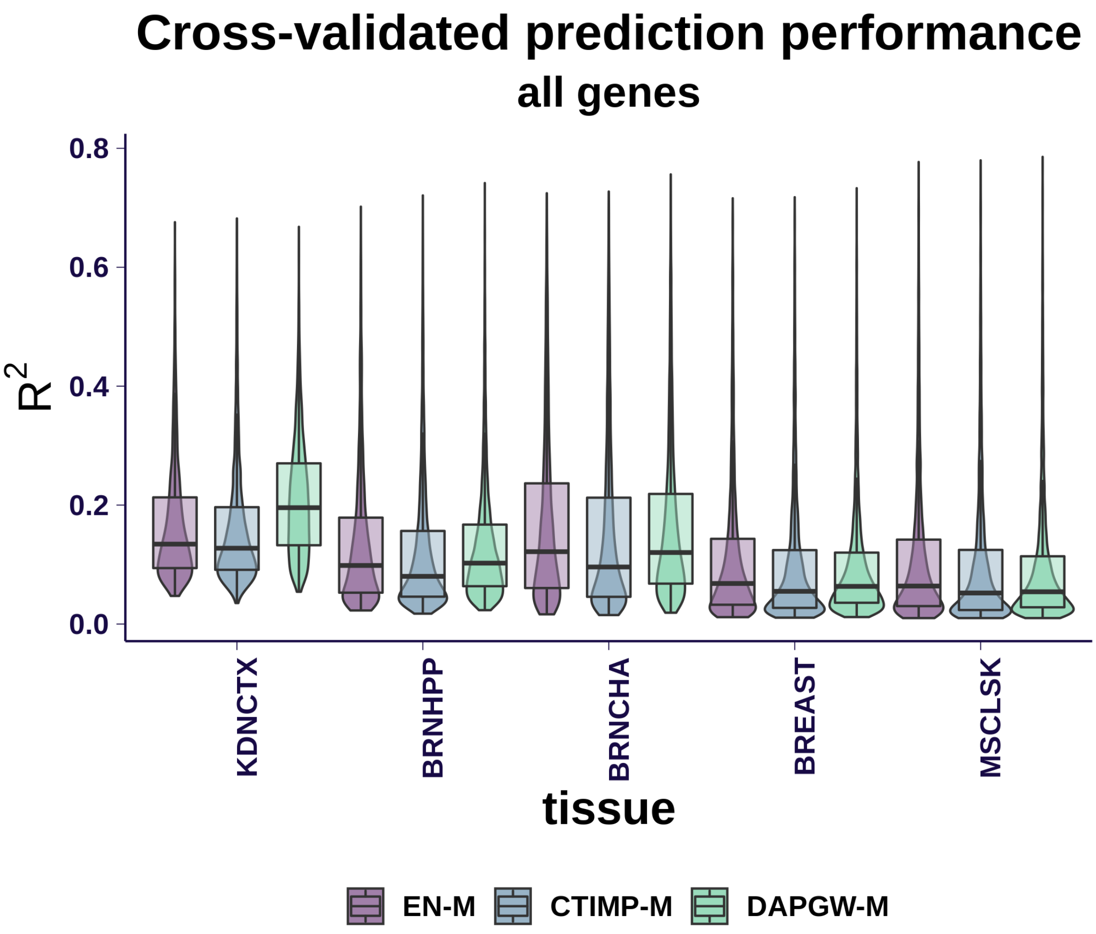

Supplementary Figure 1. Cross-validated prediction performance is shown for all available models, on sample tissues ordered from smallest sample size to largest sample size. As sample size increased, we observed similar performances on the strategies shown.
DAPGW-M is presented for illustration purposes; since it included an additional variable selection step using the same underlying data, it cannot be fairly compared to EN-M and CTIMP-M.
We note that EN-M models produced the smallest number of models (Fig. 1-A), and 82% of them are in the intersection of models available to all strategies. These tend to be genes with higher heritability and thus easier to predict. In other words, EN-M models are generally available to CTIMP-M and DAPGW-M, and the intersection of all strategies is dominated by genes in EN-M. On the other hand, CTIMP-M and DAPGW-M yield viable models for additional genes that are harder to predict, where EN-M couldn't converge to a proper model. In conclusion, CTIMP-M's and DAPGW-M's performance summary on all available genes was penalized by their convergence on genes with less signal or more complicated expression patterns.

Tissue abbreviations and sample size: KDNCTX: Kidney - Cortex, n=65; BRNHPP: Brain - Hippocampus, n=150; BRNCHA: Brain - Cerebellum, n=188; BREAST: Breast - Mammary Tissue, n=337; MSCLSK: Muscle - Skeletal, n=602

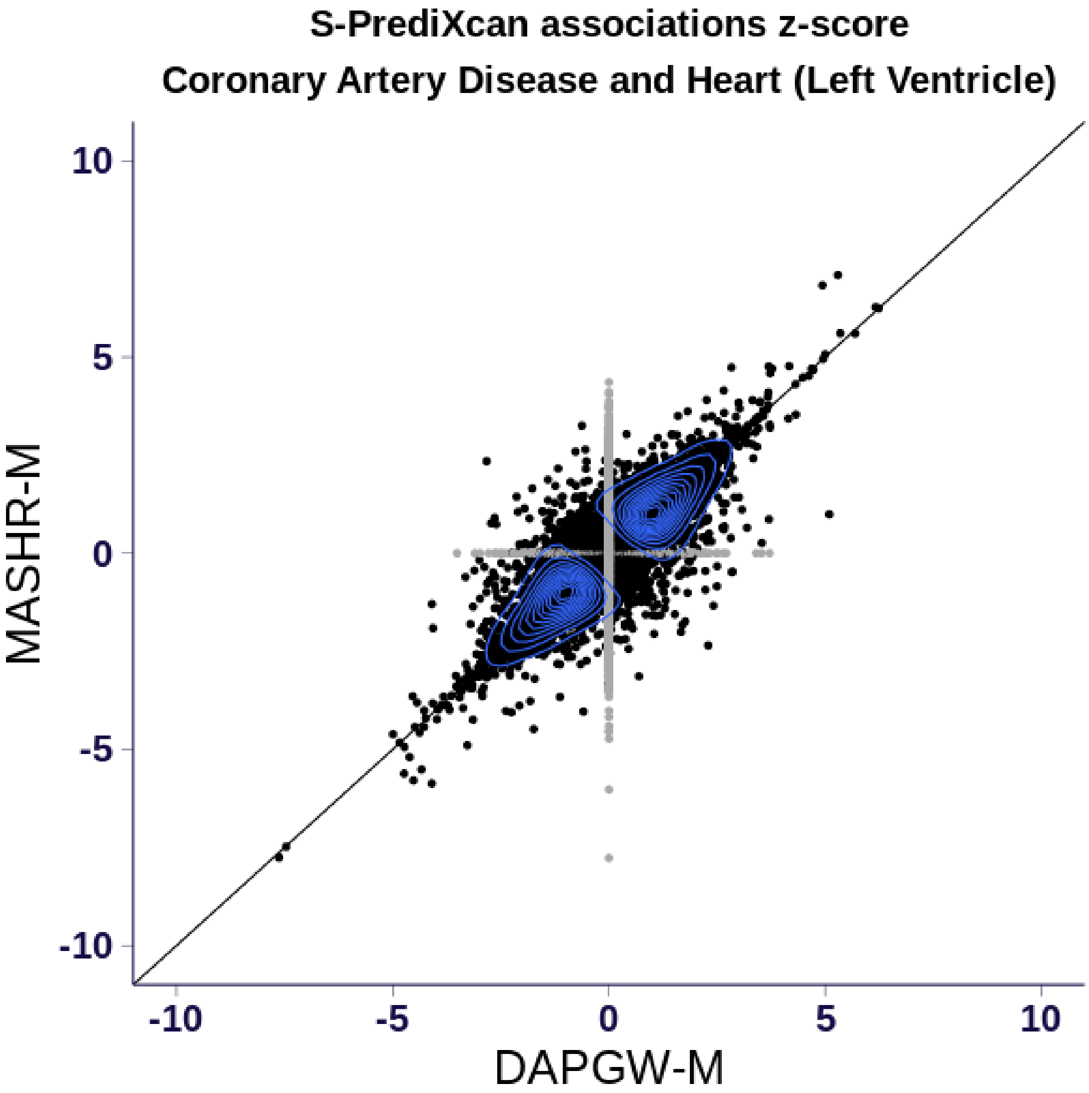

Supplementary Figure 2. This figure compares S-PrediXcan associations for Coronary Artery Disease using Left Ventricle prediction models from DAPGW-M and MASHR-M. Black dots are associations present in both DAPGW-M and MASHR-M models, while gray dots are associations present in only one of them. For shared genes, both models tend to agree in association direction and magnitude. The concordance is higher for significant associations, while poorly associated genes might even disagree in direction.
This behavior is common to all analyzed trait-tissue pairs.

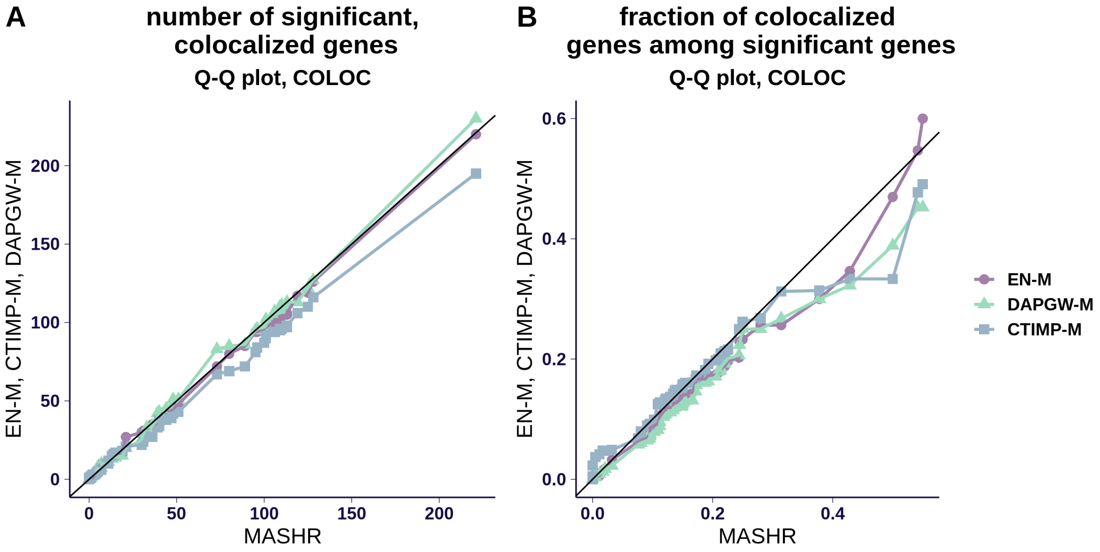

Supplementary Figure 3. This figure compares the number of S-PrediXcan associations achieving *coloc*'s probability of colocalization PP4 > 0.5.
In **panel A**, the number of detections based on *coloc* doesn't clearly distinguish between the four methods. This is likely caused by *coloc*'s assumption of only one causal variant: since genes selected by *coloc* tend to have less allelic heterogeneity, they behave similarly across the four model strategies.
In **panel B**, we observe that the fraction of significant genes that are also colocalized is slightly better for MASHR-M in general.

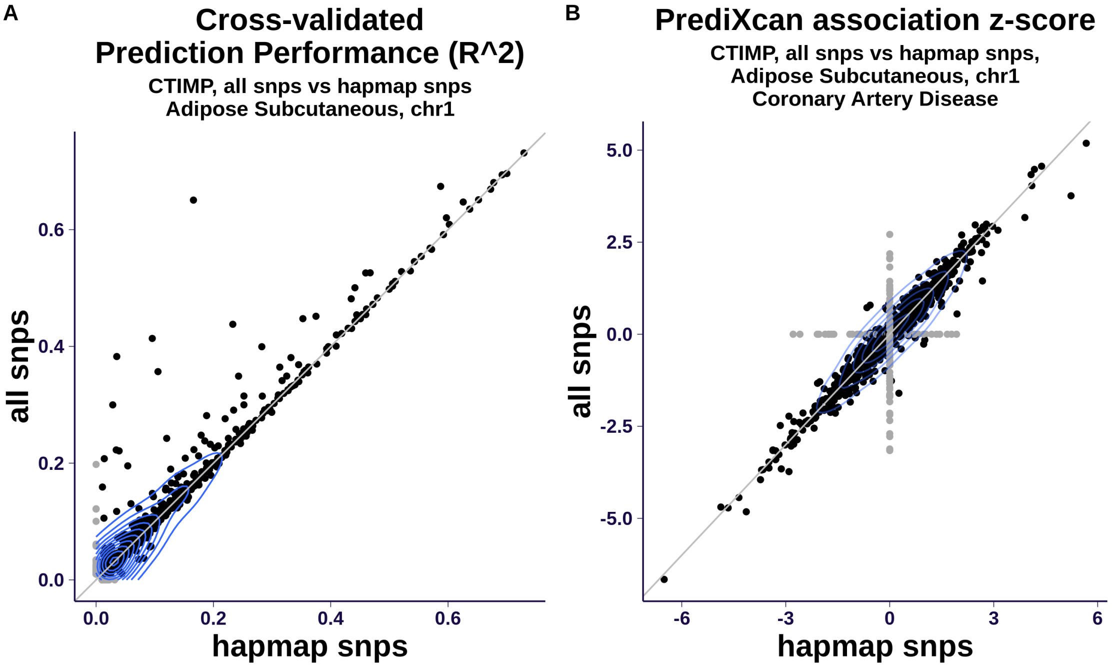

Supplementary Figure 4. CTIMP Models using all variants (CTIMP-M-AS) vs HapMap variants (CTIMP-M).
Panel A shows cross-validated prediction performance R^2. When both methods converge, they tend to achieve similar prediction performances, with CTIMP-M-AS doing slightly better on some genes.
Panel B compares S-PrediXcan associations for CTIMP-M and CTIMP-M-AS, showing a high level of agreement.

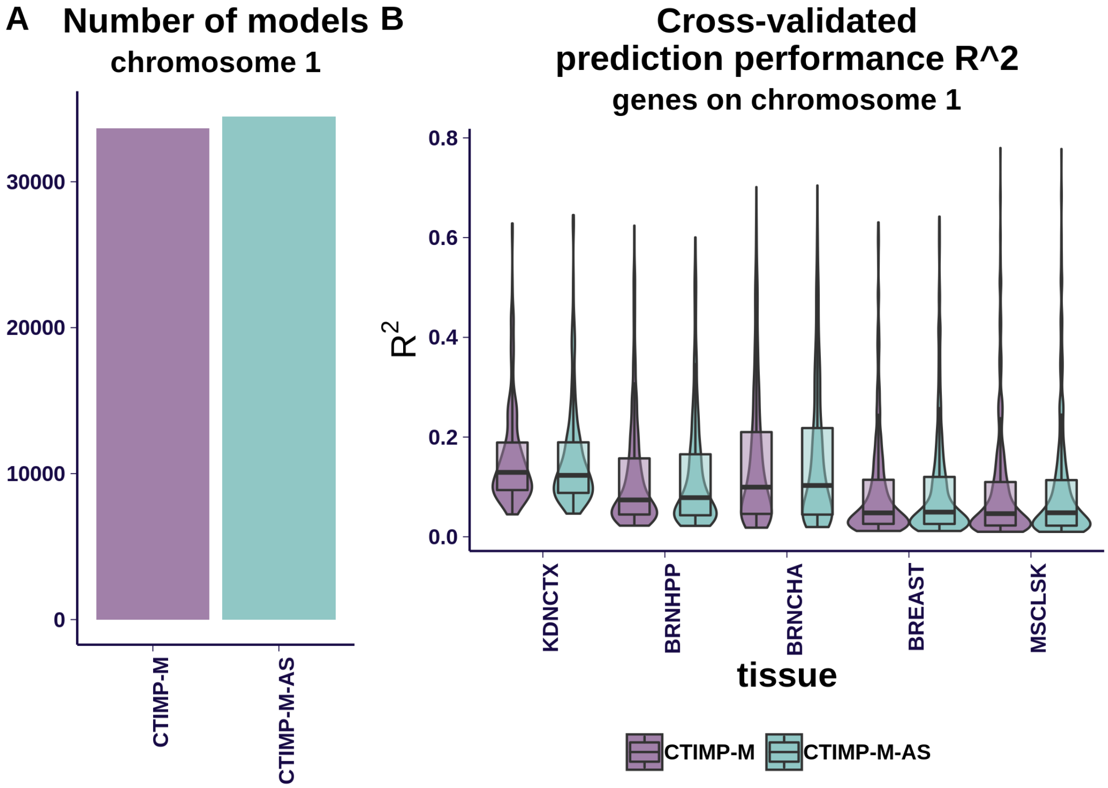

Supplementary Figure 5. CTIMP Models summary. We compare here CTIMP using all variants (abbreviated as CTIMP-M-AS) to CTIMP using only HapMap variants (abbreviated as CTIMP-M), for genes in chromosome 1. We observed no significant difference between CTIMP-M and CTIMP-M-AS. **Panel A** shows the number of generated models for protein coding genes, pseudo genes and lncRNA. **Panel B** compares prediction performance for all gene-tissue pairs, for sample tissues (ordered from smallest sample size to largest sample size). The differences in performance are negligible.

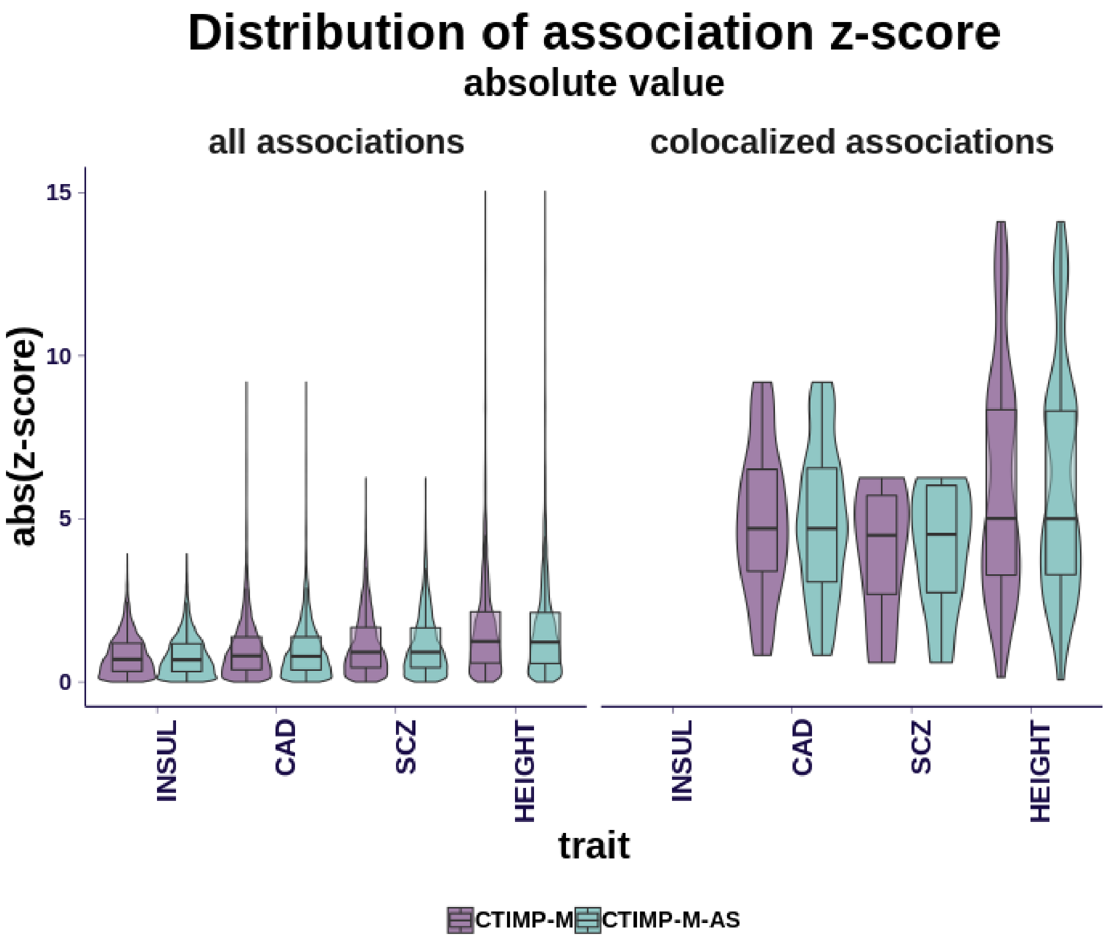

Supplementary Figure 6. CTIMP's PrediXcan associations for 4 sample traits from CTIMP models using all variants (abbreviated as CTIMP-M-AS) to CTIMP using only HapMap variants (abbreviated as CTIMP-M), for genes in chromosome 1. Both panels show absolute values of association z-score for gene-tissue pairs, to Fasting Insulin (INSUL), Coronary Artery Disease (CAD), Schizophrenia (SCZ) and Height (HEIGHT); traits are ordered from lowest to highest number of uniquely associated genes. The left panel shows all associations, whereas the right panel shows colocalized associations. We observed no significant difference between CTIMP-M and CTIMP-M-AS.
